# Investigating Major Recurring *Campylobacter jejuni* Lineages in Luxembourg Using Four Core or Whole Genome Sequencing Typing Schemes

**DOI:** 10.3389/fcimb.2020.608020

**Published:** 2021-01-08

**Authors:** Morgane Nennig, Ann-Katrin Llarena, Malte Herold, Joël Mossong, Christian Penny, Serge Losch, Odile Tresse, Catherine Ragimbeau

**Affiliations:** ^1^ Epidemiology and Microbial Genomics, Laboratoire National de Santé, Dudelange, Luxembourg; ^2^ INRAE, Oniris, SECALIM, Nantes, France; ^3^ Faculty of Veterinary Medicine, Norwegian University of Life Sciences, Oslo, Norway; ^4^ Luxembourg Institute of Science and Technology, Environmental Research and Innovation Department, Belvaux, Luxembourg; ^5^ Laboratoire de Médecine Vétérinaire de l’Etat, Veterinary Services Administration, Dudelange, Luxembourg

**Keywords:** whole genome sequencing, *Campylobacter jejuni*, typing schemes, WGS typing scheme comparison, recurring genotypes, clones, core genome MLST, whole genome MLST

## Abstract

*Campylobacter jejuni* is the leading cause of bacterial gastroenteritis, which has motivated the monitoring of genetic profiles circulating in Luxembourg since 13 years. From our integrated surveillance using a genotyping strategy based on an extended MLST scheme including *gyrA* and *porA* markers, an unexpected endemic pattern was discovered in the temporal distribution of genotypes. We aimed to test the hypothesis of stable lineages occurrence by implementing whole genome sequencing (WGS) associated with comprehensive and internationally validated schemes. This pilot study assessed four WGS-based typing schemes to classify a panel of 108 strains previously identified as recurrent or sporadic profiles using this in-house typing system. The strain collection included four common lineages in human infection (N = 67) initially identified from recurrent combination of ST-*gyrA*-*porA* alleles also detected in non-human samples: veterinary (N = 19), food (N = 20), and environmental (N = 2) sources. An additional set of 19 strains belonging to sporadic profiles completed the tested panel. All the strains were processed by WGS by using Illumina technologies and by applying stringent criteria for filtering sequencing data; we ensure robustness in our genomic comparison. Four typing schemes were applied to classify the strains: (i) the cgMLST SeqSphere+ scheme of 637 loci, (ii) the cgMLST Oxford scheme of 1,343 loci, (iii) the cgMLST INNUENDO scheme of 678 loci, and (iv) the wgMLST INNUENDO scheme of 2,795 loci. A high concordance between the typing schemes was determined by comparing the calculated adjusted Wallace coefficients. After quality control and analyses with these four typing schemes, 60 strains were confirmed as members of the four recurrent lineages regardless of the method used (N = 32, 12, 7, and 9, respectively). Our results indicate that, regardless of the typing scheme used, epidemic or endemic signals were detected as reflected by lineage B (ST2254-*gyrA*9-*porA*1) in 2014 or lineage A (ST19-*gyrA*8-*porA*7), respectively. These findings support the clonal expansion of stable genomes in *Campylobacter* population exhibiting a multi-host profile and accounting for the majority of clinical strains isolated over a decade. Such recurring genotypes suggest persistence in reservoirs, sources or environment, emphasizing the need to investigate their survival strategy in greater depth.

## Introduction

*Campylobacter* spp. is the leading cause of bacterial foodborne diarrheal disease worldwide (WHO, 2013) and the main zoonotic agent in the European Union (EU) (EFSA and ECDC, 2019). In 2018, the reported EU-wide incidence of campylobacteriosis was 64.1 cases per 100,000 population and Luxembourg had one of the highest rates in Europe (103.8) (EFSA and ECDC, 2019). *Campylobacter* is responsible for a large health and economic burden world-wide with a cost-of-illness of $1.56 billion in the USA (Scharff, 2012; Devleesschauwer et al., 2017) and 8.28 disability adjusted life years (DALYs) per 100,000 population in Europe (Cassini et al., 2018).

More than 80% of cases of campylobacteriosis are caused by *Campylobacter jejuni* and poultry is considered the main reservoir of human infections (Mughini-Gras et al., 2012; Ragimbeau et al., 2014; Mossong et al., 2016; EFSA and ECDC, 2019). Transmission is commonly associated with cross-contamination during handling of raw meat, the consumption of undercooked meat or raw drinking milk (EFSA and ECDC, 2018). *C. jejuni* lives as a commensal bacterium in the gastrointestinal tract of wild and domestic birds and mammals, including cattle and sheep. Environmental transmission routes are less frequently reported, but risks include exposure during outdoor sports, swimming in natural waters or contact with garden soil (Stuart et al., 2010; Ellis-Iversen et al., 2012; Mughini-Gras et al., 2012; Bronowski et al., 2014; Mossong et al., 2016; Kuhn et al., 2018).

Unlike for other foodborne pathogens, molecular surveillance of *C. jejuni* has not been implemented in many European countries as the majority of human infections are thought to be sporadic with a low fatality rate (0.03% in EU in 2017) (EFSA and ECDC, 2019). Nevertheless, due to the high number of reported human cases in the EU, campylobacteriosis ranks third in cause of death behind listeriosis and salmonellosis. In addition, outbreaks caused by *Campylobacter* spp. are increasingly being identified and reported on a regular basis, often linked to consumption of untreated drinking water, raw milk or chicken liver paté (Jakopanec et al., 2008; Revez et al., 2014; Davis et al., 2016; Lahti et al., 2017; Kang et al., 2019; Hyllestad et al., 2020).

The generally high incidence recorded in Luxembourg over the last decade has motivated a national implementation of molecular monitoring of *Campylobacter* circulating in food, farm animals, and environmental waters, as part of an integrated surveillance (Ragimbeau et al., 2008; Berthe et al., 2013; Ragimbeau et al., 2014; Mossong et al., 2016). Monitoring the *C. jejuni* population circulating in a community can function as early warning signals for outbreaks and detect long-term changes in the bacterial population, such as emerging new virulence traits or antimicrobial resistance. Further, monitoring the types of *C. jejuni* in different reservoirs and environments can shed light on the epidemiology of campylobacteriosis in that region.

Initially, genotypes from the molecular monitoring were defined according to an in-house typing system originally developed for the Sanger sequencing method. This typing method consists of the seven housekeeping genes from the Multi Locus Sequence Typing (MLST) method (Maiden et al., 1998; Dingle et al., 2001) combined with allelic profiles from two additional loci: *porA* (Clark et al., 2007) and *gyrA* (Wang et al., 1993). Including *porA* and *gyrA* refines the resolution scale of MLST and creates a reliable extended MLST typing method. The *porA* locus encodes the major outer membrane protein and is highly polymorphic, but stable during human passage and within family outbreaks making it a suitable molecular marker for epidemiologic investigations (Cody et al., 2009). Jay-Russell et al. (2013) supported this finding by utilizing variations in *porA* sequences as a screening tool for discriminating genetically related strains in the situation of a large outbreak (Jay-Russell et al., 2013). Interestingly, specific point mutations within *porA* were identified as markers of hyper virulence for a *C. jejuni* clone causing abortion in ruminants and foodborne disease in humans (Sahin et al., 2012; Wu et al., 2016). A sequence-based *gyrA* method was recently developed and it provides information of isolates in two respects: (i) to distinguish the major nucleotide mutation (C257T) conferring the quinolone resistance (i.e., the peptide shift Thr86Ile), and (ii) to source-track clinical isolates according to a host signature in *gyrA* alleles, potentially predictive of domestic birds as source (Jesse et al., 2006; Ragimbeau et al., 2014). The discriminative power resulting from this extended MLST method indexed on a 9-loci basis is sufficient to define different lineages and human clusters (Dingle et al., 2008; Ragimbeau et al., 2014). This has recently been superseded by whole genome sequencing (WGS).

The advent of Next Generation Sequencing (NGS) technologies has significantly increased the amount of genetic information available for the characterization of bacterial isolates. Comparisons at the genome level are more relevant for defining relationships between isolates at unprecedented resolution while simultaneously allowing the full characterization of the virulome, resistome, and metabolome of the isolate. Phylogenetic approaches based on WGS data rely on calculating genetic distances based on either SNPs (single nucleotide polymorphism) or allele differences (ADs) [known as core or whole genome MLST (cg/wgMLST)] (ECDC, 2016). Unlike other common food and waterborne bacterial pathogens [*Listeria monocytogenes* (Ragon et al., 2008) or *Salmonella enterica serovar Typhi* (Lan et al., 2009)], *Campylobacter* populations display high genetic diversity likely driven by horizontal genetic exchange (de Boer et al., 2002; Sheppard et al., 2011) and to a lesser extent by chromosomal mutations. As a result, SNP analyses that compare strains at the nucleotide level tend to overestimate genetic exchange events and, consequently, decimate the signals of the *Campylobacter* population structure (Sheppard et al., 2012). After conducting comparative studies between the SNP and the cgMLST approaches for different pathogens, it appears that the gene-by-gene approach is more suitable for identifying lineages with this recombining species (Dangel et al., 2019; Jajou et al., 2019). This gene-by-gene method defines allelic profiles from a set of common loci, known as core genome common to a representative panel of isolates. Including accessory loci, present in only a subsection of genomes and often associated with specific phenotypic traits of interest, improves the discriminatory power of the gene-by-gene analysis (Sheppard et al., 2012). For WGS analysis of *C. jejuni* and *C. coli*, several typing schemes have been developed, including two cgMLST schemes; a commercial cgMLST schema containing 637 loci from the SeqSphere+ software (Ridom GmbH, Münster, Germany; www.cgMLST.org) and the Oxford cgMLST schema with 1,343 loci (Cody et al., 2017). Two wgMLST schemes were also defined for *C. jejuni/coli* within the SeqSphere+ software (including the cgMLST and 958 accessory loci) and by the Oxford University (1,643 loci) (Cody et al., 2013). Moreover, two typing schemes were developed specifically for *C. jejuni*: a cgMLST (678 loci) and a wgMLST (2,795 loci) from the INNUENDO platform (Llarena et al., 2018). The method-dependent definition of a WGS-based genotype underlines the need for an international nomenclature to improve communication in outbreak investigation and in surveillance.

Through vigilant surveillance and molecular subtyping with extended MLST, we discovered an unexpected endemic pattern in the temporal distribution of genotypes associated with human infection over several years. The aim of this study was to investigate if these strains were indeed clonal by applying a higher resolution typing method, namely the WGS gene-by-gene approach. We simultaneously assessed the concordance between the four different typing schemes developed for *Campylobacter* spp. and their ability to separate closely related strains.

## Materials and Methods

### Strain Selection

Five thousand *C. jejuni* isolates, from human and non-human sources collected in Luxembourg between 2006 and 2018, were inspected. Years 2009 and 2010 were not included as no molecular surveillance data were available. Genotypic data associated with this collection included extended MLST profiles indexed on nine loci: 7 targets of MLST (Dingle et al., 2001), the partial sequence of *gyrA* (Ragimbeau et al., 2014), and the Sequence Variable Region of *porA* (Campylobacter MOMP database; Dingle et al., 2008). The nomenclature for displaying the results of this extended MLST was defined as follows: sequence type (ST), *gyrA* (allele number), and *porA* (allele number). For example, the combination of alleles including ST19 associated with *gyrA* allele number 8 and *porA* allele number 7 is displayed as follows: ST 19-8-7.

From these, a panel of strains with identical ST-*gyrA*-*porA* profiles over four successive years was selected, including some strains with one allele variation in either the *gyrA* or *porA* loci. Care was taken to achieve a representative strain collection from all available sources (clinical, food, animal, and environmental) and years (between 2006 and 2018). Finally, we also selected a control panel of “sporadic” isolates from patients lacking a recent travel history, i.e., only domestic cases, and whose ST-*gyrA-porA* profile occurred only once between 2011 and 2018. This control panel was used as outgroup.

### Culture, DNA Extraction, Library Preparation, and WGS

All isolates were stored in −80°C in FBP medium (a combination of ferrous sulfate, sodium metabisulfite, sodium pyruvate and glycerol) (Gorman and Adley, 2004). For each strain, a loopful of frozen culture was spread on chocolate PolyVitex plates (BioMerieux, Marcy-l’Etoile, France) and incubated under micro-aerobic conditions (5% O_2_, 10% CO_2_, 85% N_2_) at 42°C for 48 h. Then, a subculture of one colony was made again on chocolate PolyVitex agar, and incubated 16 h in the above-mentioned conditions. DNA was extracted with the DNA QIAamp Mini Kit (Qiagen, The Netherlands) according to the manufacturer’s instructions. DNA was quantified with the Qubit 2.0 Fluorometer (Invitrogen, Belgium) and the Qubit^®^ dsDNA BR Assay Kit (Life Technologies, Belgium). The DNA concentration was adjusted to be within the range of 30 to 170 µg/ml for subsequent sequencing. Libraries were prepared using the Nextera™ DNA Flex Library Prep Kit or the Nextera™ XT DNA Library Preparation Kit and sequenced on the MiSeq or the MiniSeq platforms achieving either 150- or 250-bp paired-end reads. All chemistry and instrumentations are supplied by Illumina, San Diego, CA, USA. Sequenced raw reads have been uploaded to ENA and are available under the accession project number PRJEB40465.

### Genome Assembly and Quality Control (QC) Criteria

For the cgMLSTs SeqSphere+ and Oxford, the paired-end raw read data were *de novo* assembled using Velvet Optimizer v.1.1.04 implemented in Ridom SeqSphere+ v6.1 (Ridom GmbH, Münster, Germany) (Jünemann et al., 2013). Velvet Optimizer was run with automatic determination of the coverage cut-off and minimum contig length and only assemblies with >30x coverage, 1.6Mb ± 10% bp in size and maximum number of 150 contigs were included in the downstream analysis (Zerbino and Birney, 2008; Cody et al., 2017). For the cgMLST INNUENDO and wgMLST INNUENDO, the raw data were assembled into contigs using the INNUca pipeline v. 4.2.1 with default settings (Machado et al. 2017). Only profiles with no more than 2% of missing loci in either cgMLST were included in the comparative study.

### WGS-Based Typing Schemes for Genome Comparison

#### cgMLST and Accessory Schemes in SeqSphere+

For SeqSphere+, an *ad hoc* cgMLST scheme (N = 637 loci) for *C. jejuni/C. coli* developed by the commercial firm Ridom SeqSphere+ and publicly available at www.cgMLST.org was used. Details of the material and methods used for defining this typing scheme were kindly provided by Prof. Dr. Harmsen (**Supplementary Data S1**). The cgMLST scheme consisted of 637 genes (https://www.cgmlst.org/ncs/schema/145039/locus/). Using genomic data from previously described local outbreaks, a Complex Type (CT) threshold of thirteen was defined to give guidance for delineation of possibly related from not-related genomes (Mellmann et al., 2004). In addition, cgMLST (v1.3) was merged with a screening of the alleles of the accessory genes (N = 958). Altogether, the combined typing wgMLST scheme targets 1,595 loci and the nomenclature remain the same as in the cgMLST analyses with the definition of CTs, solely based on core genome analyses, with a cluster alert of 13.

#### cgMLST Oxford Scheme

Cody et al. (2017) designed a cgMLST scheme composed of 1,343 loci, available as an open-access and web-accessible analyses online (PubMLST - Campylobacter Sequence Typing; Jolley et al., 2018). The system assigns a unique profile ID from each isolate sequences submitted. Clustering to identify groups can be performed by selecting a threshold empirically chosen (depending on the discrimination power needed). However for this study, the scheme was implemented in SeqSphere+ for comparing strains by using an in-house nomenclature.

#### cgMLST and wgMLST INNUENDO Schemes

The cgMLST and wgMLST schemes from INNUENDO include 678 and 2,795 loci, respectively, and are publicly available at Zenodo (https://zenodo.org/record/1322564#.X5l_4IhKg2y, Rossi et al., 2018). The cgMLST and wgMLST profiles of the INNUca assembled genomes produced in this study were called using chewBBACA suite (v 2.0.17.1) (Silva et al., 2018). The achieved cgMLST profiles were added to the cgMLST allelic profiles of the 6,526 *C. jejuni* genomes of the INNUENDO dataset, which is also available at Zenodo (Allele_Profiles/Cjejuni_cgMLST_alleleProfiles.tsv, https://zenodo.org/record/1322564#.X5l_4IhKg2y, Rossi et al., 2018). Minimum Spanning Trees (MST) and goeBURST distances were calculated using the goeBURST Full MST algorithm implemented in PHYLOViZ 2.0, and used to define L1:L2:L3 profiles for the cgMLST at 4, 59, and 292 loci variance (Feil et al., 2004; Francisco et al., 2009; Francisco et al., 2012; Nascimento et al., 2017; Llarena et al., 2018). This classification system is hierarchical: L1 is the level representing the highest resolution with a threshold of 4 and it is applied for outbreak detection and investigation, L2 is the intermediate level and is used for long-term longitudinal monitoring. L3 is defined as the level with the highest concordance with the seven-gene MLST classification (Llarena et al., 2018). The wgMLST INNUENDO defines genotypes based on the combination of alleles from the 2,795 loci; no rules were initially developed for clustering isolates with similar profiles.

### Comparison of the Targets Included in Each cgMLST Schemes

To crosslink loci with different naming conventions across the four typing schemes, we compared the allele sequences in a pairwise manner. Allele sequences for cgMLST SeqSphere+ were downloaded from https://www.cgmlst.org/ncs/schema/145039/. Allele sequences for cgMLST Oxford were downloaded *via* the pubMLST RESTful API (scheme 4) (Jolley et al., 2017). Allele sequences for cgMLST INNUENDO were downloaded from Zenodo (Rossi et al., 2018). We selected the first allele sequence for each loci of the four typing schemes and performed pairwise reciprocal best hit comparison for the three schemes with the rbh function of the MMseqs2 toolkit ver. 11.e1a1c (Mirdita et al., 2019) using nucleotide search including forward and reverse strand, as well as default parameters. Hits with bitscore above 100 were selected and connected across schemes with a custom script in R 3.4.4. (R Core Team, 2018) using the igraph package 1.2.5. (Csárdi and Nepusz, 2006). Sets of matching loci within the three schemes were visualized with the UpSetR 1.4.0. package (Conway et al., 2017).

### Typing System Concordance

The adjusted Wallace coefficient (AWC) (Wallace, 1983; Severiano et al., 2011) was used to estimate the concordance between the different typing schemes in classifying strains (Pinto et al., 2008) by the online Comparing Partitions tool (http://www.comparingpartitions.info), using the strain panel (**Supplementary Data S2**). The degree of equivalence is reflected by AWC. It indicates the probability that two strains with the same type by one method are also categorized into the same type by another method.

### Detection of wgMLST Targets Shared by Recurrent Lineages

To determine the overlap of detected wgMLST INNUENDO targets, the allelic profiles of all strains were compared. We extracted lists of targets that appeared at least once within each of the respective lineages in the collection of strains to determine and visualize overlapping and unique sets with a webtool (http://www.molbiotools.com/listcompare.html).

### Cluster Analyses

In SeqSphere+, *Campylobacter* isolates are classified in CTs in which the first CT assigned chronologically is definitively fixed in the database and referred to as the CT founder (Ridom SeqSphere+, 2013). In contrast, the goeBURST algorithm produces a hierarchical classification with the gene-by-gene approach and aims to predict the founder of a clonal complex based on the allele frequency in the dataset. It assumes that the ancestral genotype is the predominant one, which subsequently generates variants. To deduct and visualize, the possible evolutionary relationships between strains, the goeBURST algorithm and its expansion to generate a complete MST implemented in PHYLOViZ 2.0 was used for the cgMLSTs SeqSphere+, INNUENDO, Oxford, the cgMLST SeqSphere+ combined with the accessory targets and the wgMLST INNUENDO (https://online2.phyloviz.net/index) (Feil et al., 2004; Nascimento et al., 2017).

The dynamic shared-genome based approach was performed on the MST generated for the cgMLST Oxford and the wgMLST INNUENDO in order to determine a clustering threshold. Genomic clusters were determined according to the definition of goeBURST groups, based on allelic differences ranging from 0.5 to 1% (Llarena et al., 2018). The in-house nomenclature for displaying the results of cgMLST Oxford and wgMLST INNUENDO were defined as follows: Ox+number and wg+number. For example, the Ox profile number 10 and the wg profile number 8 are displayed as follows: Ox10 and wg8, respectively. The wgMLST profiles were used in the dynamic shared-genome based approach for the comparison and only to increase resolution for clustering strains.

## Results

### Recurrent Extended MLST Profiles in Campylobacteriosis

**Figure 1:**
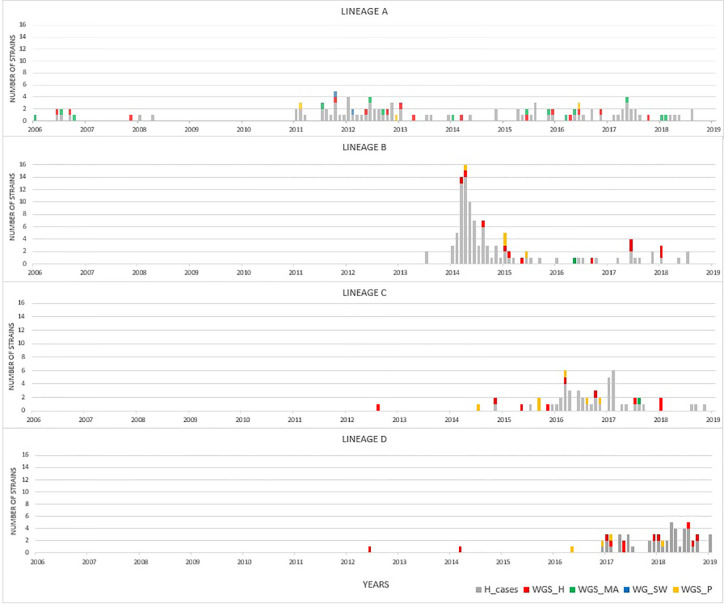
Distribution of strains occurrence for lineages A to D over time. Clinical strains of the laboratory collection are displayed in gray (extended MLST typing). Colors represent to source of selected isolates that were analyzed by WGS: human (red), cattle and sheep (green), poultry (yellow), and surface water (blue) samples.

By focusing solely on human clinical isolates from our historical collection (N = 3,000), we identified approximately one hundred distinct ST-*gyrA*-*porA* combinations. Two-thirds (N = 2,010) of the human strains in the collection belong to 108 main combinations (**Supplementary Data S3**). Four lineages (ST19-8-7, ST2254-9-1, ST464-8-1678, and ST6175-9-1625, hereafter referred to as lineage A, B, C, and D, respectively) were selected due to the high number of strains (N ≥ 45) and their frequency in human infection over time (**Figure 1** and **Supplementary Data S3**). Some minor variations were accepted in *gyrA* and *porA* alleles: three variants of *porA* and one of *gyrA* in lineage A, and one variant of *porA* in lineage B (**Table 1**). In lineage A, the variation of *gyrA* alleles (*gyrA*1 instead of *gyrA*8) leads to the loss of the quinolone resistance (Wang et al., 1993; Payot et al., 2006). Concerning the *porA* variations, two are linked to deletions in lineage A and two to a non-synonymous mutation (one in lineage A and one in lineage B, respectively). Lineage A has appeared regularly after 2005, with an average of five strains per year and up to 23 in 2012, while 68% of all the strains belonging to lineage B were gathered in a peak in 2014 (**Figure 1**). For lineage C, strains displayed the same combination of alleles and occurred once in August 2008 and then reemerged from July 2014 to January 2018 (**Figure 1**). For lineage D, strains were characterized by the same allele combination (**Table 1**) and occurred once in June 2012, once in March 2014 and then regularly, from May 2016 to October 2018 (**Figure 1**).

**Table 1:** Distribution of main lineages, extended MLST, and variant of the strain collection.

Lineage	Main combination	Variants	Human	Poultry	Ruminants	Environmental	Total
A	ST 19 – *gyrA* 8 – *porA* 7	ST 19 – *gyrA* 8 – *porA* 582 ST 19 – *gyrA* 8 – *porA* 2070 ST 19 – *gyrA* 8 – *porA* 2068 ST 19 – *gyrA* 1 – *porA* 7	13 1 1 1	3 1	14 1	2	37
B	ST 2254 – *gyrA* 9 – *porA* 1	ST 2254 – *gyrA* 9 – *porA* 275	10 1	4	1	0	16
C	ST 464 – *gyrA* 8 – *porA* 1678		10	8	1	0	19
D	ST 6175 – *gyrA* 9 – *porA* 1628		12	4	1	0	17
Total			49	20	18	2	89

ST, Sequence Type.

### Selection of a Strain Panel

Overall, the selected panel included strains from various sources as the four lineages occurring in human infections were also detected in non-human samples. Altogether, the collection included isolates from human (N = 67), poultry (N = 21), and ruminant (N = 18). To complete the panel, two strains from environmental sources (surface waters) assigned to lineage A were added (**Table 1**). A total of 108 strains was selected for the strain panel and subjected to WGS. To achieve equal distribution of strains over the study period, strains belonging to lineage A (N = 37 of 70), lineage B (N = 16 of 97), lineage C (N = 19 of 45), and lineage D (N = 17 of 58) were selected. In addition, 19 strains with a unique ST-*gyrA*-*porA* combination were included in the panel as an outgroup (**Supplementary Data S2**).

The acquired assemblies varied between 35× and 120× in depth of coverage and 1 to 150 contigs, associated with a percentage of good targets ranging from 98.6% to 99.8% (mean value = 99.3%) for cgMLST SeqSphere+ and from 98.0% to 99.3% (mean value = 98.4%) for the cgMLST Oxford. According to the quality criteria defined above (see Methods 2.3) as well as those of the INNUca pipeline, 15 genomes were discarded (14 with SeqSphere+ and 1 with INNUENDO criteria; 4, 1, 4 and 7 genomes were removed from lineages A, B, C and D, respectively). Consequently, genomes of 93 strains were included in the downstream analysis (**Supplementary Data S2**).

### Comparison of the Loci Included in the Different Schemes

**Figure 2:**
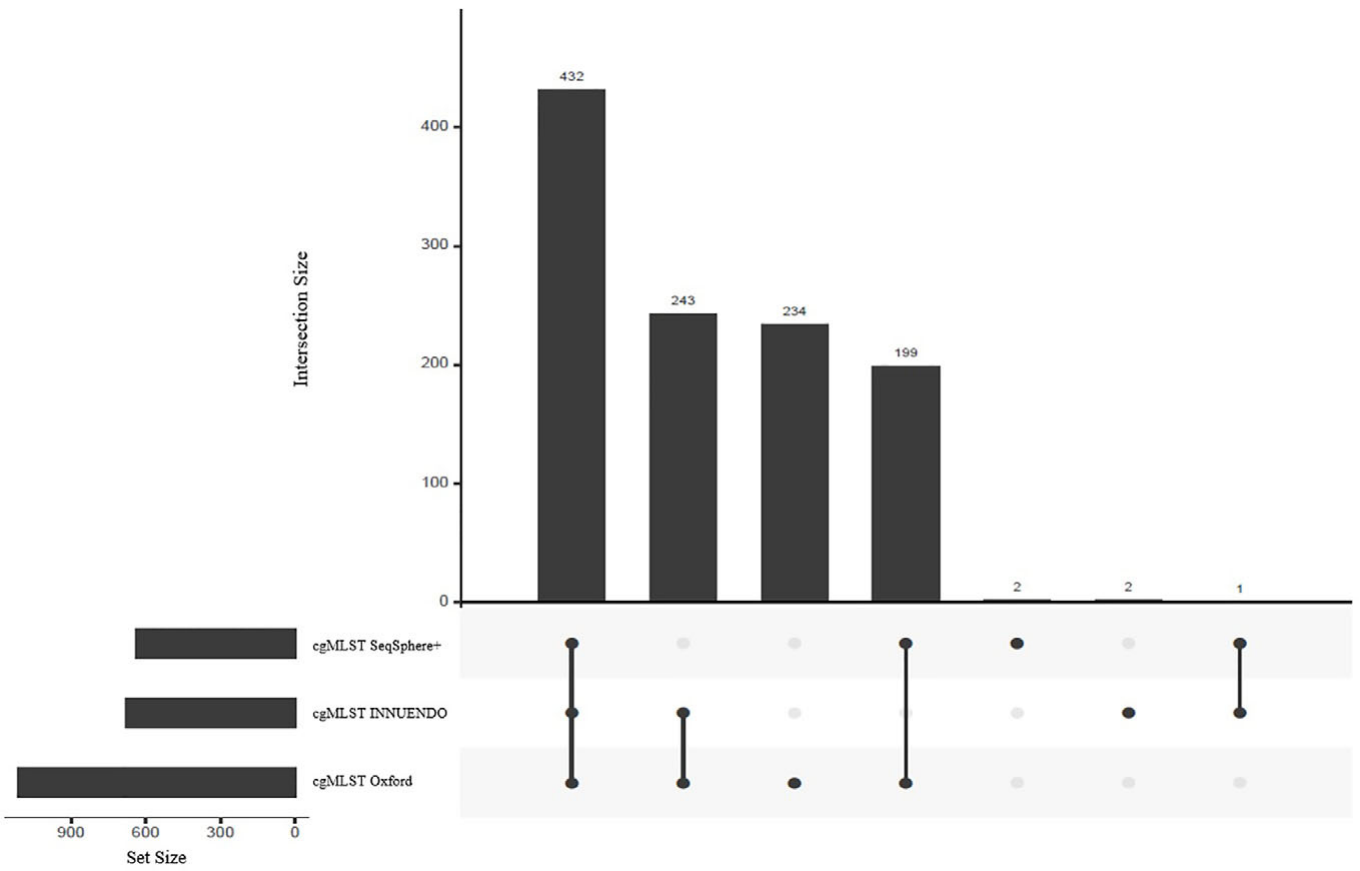
Shared targets between the three compared schemes: cgMLST SeqSphere+ (637 targets), cgMLST INNUENDO (678 targets) and cgMLST Oxford (1,343 targets) highlighted as set sizes. The central bars represent the number of shared or unique targets in or between the different schemes. The points below define the members of the respective sets. For example, 432 targets are present in all three cgMLSTs (SeqSphere+, Oxford, and INNUENDO) and 243 targets are present in both the cgMLSTs Oxford and INNUENDO but not in the cgMLST SeqSphere+. For an overview of shared targets, also refer to **Supplementary Data S4**.

As the number of loci selected for the core genome varies between the schemes, we compared the respective sequences to assess the number of shared loci. We compared allele sequences by reciprocal best hits. All schemes shared 432 loci, constituting the majority of targets in cgMLST SeqSphere+ and cgMLST INNUENDO with 68% and 64% of targets respectively (**Figure 2**). The majority of targets that differed between cgMLST SeqSphere+ and cgMLST INNUENDO was present in cgMLST Oxford. The wgMLST INNUENDO had an additional 1,775 loci not present in any of the other three cgMLST schemas (**Supplementary Data S4**). The mean size of targets included in each cgMLST typing scheme ranges from 93 to 4,553 bp and the complete lists of targets are provided in **Supplementary Data S4**.

### Gene-by-Gene WGS Analysis

**Figure 3:**
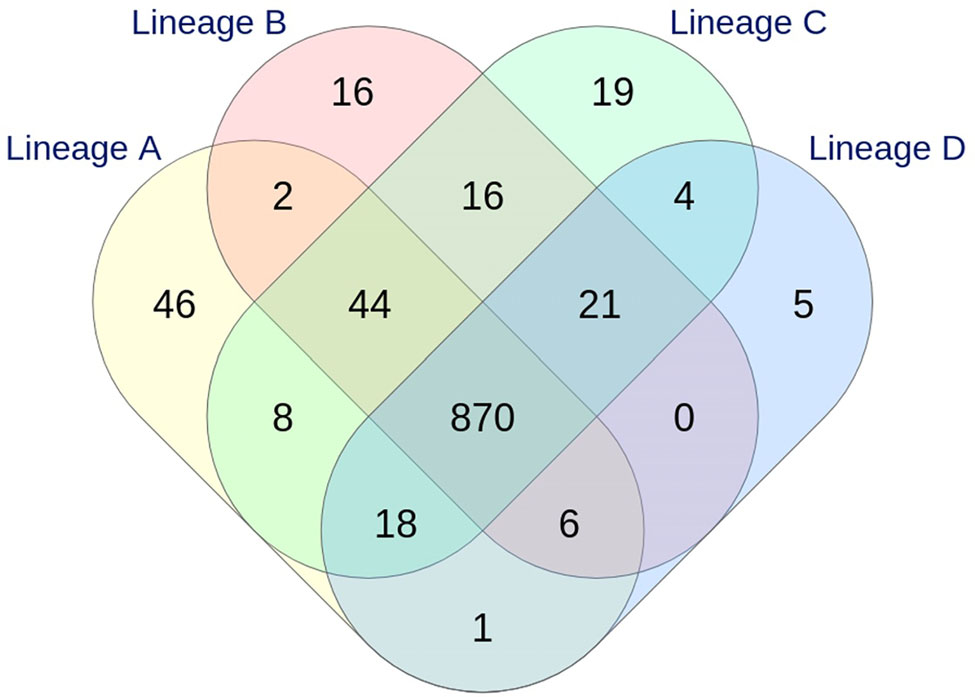
Venn diagram showing the relationship between the loci identified in the four lineages by wgMLST INNUENDO analysis (2,795 targets). A total of 995, 975, 1,000, and 925 targets were detected in lineages A, B, C, and D, respectively.

With the dynamic shared-genome based approach using 1% allelic differences, thresholds of 11 and 9 AD were defined to classify the strains by the cgMLST Oxford and the wgMLST INNUENDO scheme respectively (**Table 2**). The number of partitions, or clusters, obtained with the different methods was very close: 28 for extended MLST, 22 for cgMLST SeqSphere+, 26 for cgMLST Oxford, and 24 for cgMLST INNUENDO. The largest number of partitions (N = 32) was obtained with the wgMLST INNUENDO analysis (**Supplementary Data S2**). From this pan-genome analysis including 2,795 targets, an average of 974 loci was detected in each lineage, with 870 loci shared between the four lineages (**Figure 3**).

**Table 2:** Characteristics of the different typing schemes to analyze WGS data from *C. jejuni*.

Typing scheme	Number of targets	Cluster Alert distance*
Extended MLST	9	1
cgMLST SeqSphere+	637	13
cgMLST Oxford	1,343	11
cgMLST INNUENDO	678	L1: 4, L2: 59, and L3: 292
wgMLST INNUENDO	2,795	9

*The cluster alert distance is defined by a threshold value corresponding to the maximum number of different alleles between strains belonging to the same cluster.

MLST, Multi Locus Sequence Typing; cg, core genome; wg, whole genome.

**Figure 4:**
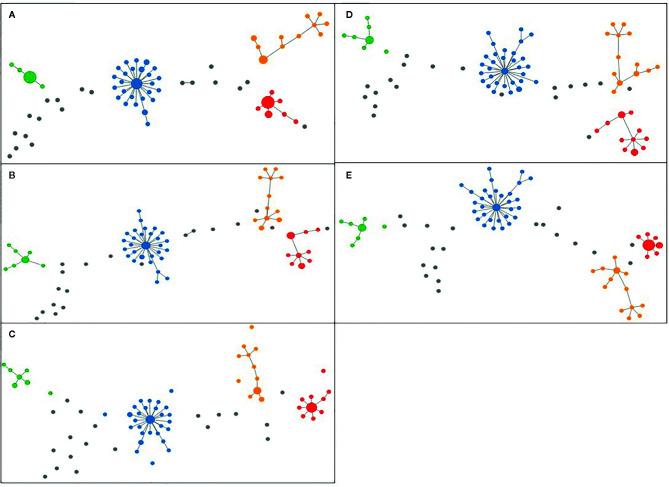
Minimum Spanning Trees generated using PHYLOViZ for **(A)** cgMLST SeqSphere+ (cut-off: 13), **(B)** cgMLST and accessory targets SeqSphere+ (cut-off: 13), **(C)** cgMLST INNUENDO (cut-off: 4), **(D)** cgMLST Oxford (cut-off defined by dynamic core analysis: 11) and **(E)** wgMLST INNUENDO (cut-off defined by dynamic core analysis: 9) analyses on tool. Lineage A is displayed in blue, lineage B in red, lineage C in orange, and lineage D in green and unique combinations in gray.

For the analysis of unique combinations, all sporadic strains were classified distinctly by the typing schemes, with one exception regarding two strains that were classified in the same CT (CT 1639) with cgMLST SeqSphere+, in the same profile L1:L2:L3 (66:81:1) with cgMLST INNUENDO and in the same profile with wgMLST INNUENDO (wg30). The allelic profiles for the strains generated by all typing methods were clustered and visualized in PHYLOViZ online tool, in which all five typing schemes achieved very similar unrooted MSTs (**Figure 4**) (PHYLOViZ Online).

Lineage A (ST19-8-7, N = 34) had very limited genetic diversity according to our gene-by-gene WGS analyses. The cgMLST SeqSphere+ assigned all strains to the same CT (CT 82), as did the cgMLST Oxford scheme: Ox1 (**Table 3**). On the contrary, the cgMLST INNUENDO divided lineage A in two groups, of which the majority (33/34) were of the same L1:L2:L3 profile (1:9:1). The 34^th^ strain had a different genotype at L1 level (2695:9:1). Thirty-two of 34 lineage A strains had an identical wgMLST INNUENDO profile: wg1, while two strains had a deviating wgMLST profile (wg5 and wg6). The strains belonging to profile wg1 were collected over a wide timespan (2006–2018) and a range of sources (human, veterinary, or environmental sources).

**Table 3:** Assignment of genetic profiles according to the different typing schemes for strains initially belonging to the lineage A.

Strain ID	Isolation Source	Year of isolation	ST – *gyrA* – *porA* – Extended MLST	CT – cgMLST SeqSphere+	Genotype – cgMLST Oxford	L1:L2:L3 – cgMLST INNUENDO	Genotype – wgMLST INNUENDO
Camp001	MA	2005	19 – 8 – 7	82	Ox1	1:9:1	wg1
Camp005	H	2006	19 – 8 – 7	82	Ox1	1:9:1	wg1
Camp003	H	2006	19 – 8 – 7	82	Ox1	1:9:1	wg1
Camp004	MA	2006	19 – 8 – 7	82	Ox1	1:9:1	wg1
Camp006	H	2007	19 – 8 – 7	82	Ox1	1:9:1	wg1
Camp002	MA	2006	19 – 8 – 7	82	Ox1	1:9:1	wg1
Camp009	H	2011	19 – 8 – 7	82	Ox1	1:9:1	wg1
Camp010	MA	2011	19 – 8 – 7	82	Ox1	1:9:1	wg1
Camp008	SW	2011	19 – 8 – 7	82	Ox1	1:9:1	wg1
Camp014	H	2012	19 – 8 – 7	82	Ox1	1:9:1	wg1
Camp012	MA	2012	19 – 8 – 7	82	Ox1	1:9:1	wg1
Camp011	SW	2012	19 – 8 – 7	82	Ox1	1:9:1	wg1
Camp015	MA	2012	19 – 8 – 7	82	Ox1	1:9:1	wg1
Camp016	V	2013	19 – 8 – 7	82	Ox1	1:9:1	wg1
Camp018	H	2013	19 – 8 – 7	82	Ox1	1:9:1	wg1
Camp017	H	2013	19 – 8 – 7	82	Ox1	1:9:1	wg1
Camp021	MA	2014	19 – 8 – 7	82	Ox1	1:9:1	wg1
Camp019	H	2014	19 – 8 – 7	82	Ox1	1:9:1	wg1
Camp020	MA	2014	19 – 8 – 7	82	Ox1	1:9:1	wg1
Camp023	MA	2015	19 – 8 – 7	82	Ox1	1:9:1	wg1
Camp029	MA	2016	19 – 8 – 7	82	Ox1	1:9:1	wg1
Camp028	H	2016	19 – 8 – 7	82	Ox1	1:9:1	wg1
Camp027	H	2016	19 – 8 – 7	82	Ox1	1:9:1	wg1
Camp032	V	2016	19 – 8 – 7	82	Ox1	1:9:1	wg1
Camp031	H	2016	19 – 8 – 7	82	Ox1	1:9:1	wg1
Camp035	H	2017	19 – 8 – 7	82	Ox1	1:9:1	wg1
Camp036	MA	2018	19 – 8 – 7	82	Ox1	1:9:1	wg1
Camp037	MA	2018	19 – 8 – 7	82	Ox1	1:9:1	wg1
Camp024	MA	2015	19 – 8 – 582	82	Ox1	1:9:1	wg1
Camp025	H	2015	19 – 8 – 2068	82	Ox1	1:9:1	wg1
Camp022	H	2015	19 – 8 – 2070	82	Ox1	1:9:1	wg1
Camp013	H	2012	19 – 8 – 7	82	Ox1	2695:9:1	wg1
Camp030	V	2016	19 – 8 – 7	82	Ox1	1:9:1	wg5
Camp034	H	2017	19 – 1 – 7	82	Ox11	1:9:1	wg6

In the column Isolation Source, H refers to clinical samples, MA to mammals (cattle and sheep), V to poultry, and SW to surface waters.

ST, Sequence Type; CT, Complex Type; MLST, Multi Locus Sequence Typing; cg, core genome; wg, whole genome.

Lineage B (ST2254-9-1) had low genetic variability according to the cg/wgMLST analyses: altogether, 15 of 16 strains had a similar cgMLST SeqSphere+ (CT 51), cgMLST Oxford (Ox2), and cgMLST INNUENDO (19:49:4) profiles. The increased resolution offered by the wgMLST INNUENDO divided the strains in three types: 75% of the strains were of wg2 while the remaining quarter was divided between wg7 and wg8. The strains belonging to the genotype wg2 were isolated from 2014 to 2018 and from diverse sources (**Table 4**).

**Table 4:** Assignment of genetic profiles according to the different typing schemes for strains initially belonging to the lineage B.

Strain ID	Isolation Source	Year of isolation	ST – *gyrA* – *porA* – Extended MLST	CT – cgMLST SeqSphere+	Genotype – cgMLST Oxford	L1:L2:L3 – cgMLST INNUENDO	Genotype – wgMLST INNUENDO
Camp038	H	2014	2254 – 9 – 1	51	Ox2	19:49:4	wg2
Camp041	V	2014	2254 – 9 – 1	51	Ox2	19:49:4	wg2
Camp040	H	2014	2254 – 9 – 1	51	Ox2	19:49:4	wg2
Camp045	V	2015	2254 – 9 – 1	51	Ox2	19:49:4	wg2
Camp043	H	2015	2254 – 9 – 1	51	Ox2	19:49:4	wg2
Camp046	V	2015	2254 – 9 – 1	51	Ox2	19:49:4	wg2
Camp042	H	2015	2254 – 9 – 1	51	Ox2	19:49:4	wg2
Camp047	H	2015	2254 – 9 – 1	51	Ox2	19:49:4	wg2
Camp044	V	2015	2254 – 9 – 1	51	Ox2	19:49:4	wg2
Camp048	H	2016	2254 – 9 – 1	51	Ox2	19:49:4	wg2
Camp051	H	2017	2254 – 9 – 1	51	Ox2	19:49:4	wg2
Camp052	H	2018	2254 – 9 – 1	51	Ox2	19:49:4	wg2
Camp049	MA	2016	2254 – 9 – 1	51	Ox2	19:49:4	wg7
Camp053	H	2018	2254 – 9 – 1	51	Ox2	19:49:4	wg7
Camp050	H	2017	2254 – 9 – 275	51	Ox2	19:49:4	wg8

In the column Isolation Source, H refers to clinical samples, MA to mammals (cattle and sheep), V to poultry, and SW to surface waters.

ST, Sequence Type; CT, Complex Type; MLST, Multi Locus Sequence Typing; cg, core genome; wg, whole genome.

Lineage C (ST464-8-1678) was more variable than A and B: all 15 strains were of the CT 75 and the 29:70:7 according to the cgMLST SeqSphere+ and cgMLST INNUENDO, respectively. Contrary to this, the cgMLST Oxford split the panel into three: Ox3, Ox5, and Ox6 (**Table 5**). The wgMLST INNUENDO discriminated six different genotypes collected from diverse range of sources between 2014 and 2017 (**Table 5**).

**Table 5:** Assignment of genetic profiles according to the different typing schemes for strains initially belonging to the lineage C.

Strain ID	Isolation Source	Year of isolation	ST – *gyrA* – *porA* – Extended MLST	CT – cgMLST SeqSphere+	Genotype – cgMLST Oxford	L1:L2:L3 – cgMLST INNUENDO	Genotype – wgMLST INNUENDO
Camp059	V	2015	464 – 8 – 1678	75	Ox3	29:70:7	wg3
Camp058	H	2015	464 – 8 – 1678	75	Ox3	29:70:7	wg3
Camp067	V	2016	464 – 8 – 1678	75	Ox3	29:70:7	wg3
Camp065	H	2016	464 – 8 – 1678	75	Ox3	29:70:7	wg3
Camp064	V	2016	464 – 8 – 1678	75	Ox3	29:70:7	wg3
Camp069	MA	2017	464 – 8 – 1678	75	Ox3	29:70:7	wg3
Camp063	V	2016	464 – 8 – 1678	75	Ox3	29:70:7	wg3
Camp060	H	2015	464 – 8 – 1678	75	Ox3	29:70:7	wg9
Camp070	H	2017	464 – 8 – 1678	75	Ox3	29:70:7	wg10
Camp055	V	2014	464 – 8 – 1678	75	Ox3	29:70:7	wg11
Camp056	H	2014	464 – 8 – 1678	75	Ox3	29:70:7	wg11
Camp066	V	2016	464 – 8 – 1678	75	Ox3	29:70:7	wg11
Camp068	H	2017	464 – 8 – 1678	75	Ox3	29:70:7	wg11
Camp054	H	2012	464 – 8 – 1678	75	Ox5	29:70:7	wg12
Camp072	H	2018	464 – 8 – 1678	75	Ox6	29:70:7	wg13

In the column Isolation Source, H refers to clinical samples, MA to mammals (cattle and sheep), V to poultry, and SW to surface waters.

ST, Sequence Type; CT, Complex Type; MLST, Multi Locus Sequence Typing; cg, core genome; wg, whole genome.

For lineage D (ST6175-9-1625), all the 10 strains were gathered by the cgMLST SeqSphere+ in the same CT (CT 543), while with the cgMLSTs Oxford and INNUENDO and the wgMLST INNUENDO, one strain had a different profile from the others. The strains were isolated between 2017 and 2018 and from diverse sources (**Table 6**).

**Table 6:** Assignment of genetic profiles according to the different typing schemes for strains initially belonging to the lineage D.

Strain ID	Isolation Source	Year of isolation	ST – *gyrA* – *porA* – Extended MLST	CT – cgMLST SeqSphere+	Genotype – cgMLST Oxford	L1:L2:L3 – cgMLST INNUENDO	Genotype – wgMLST INNUENDO
Camp082	V	2017	6175 – 9 – 1625	543	Ox4	41:68:27	wg4
Camp083	H	2017	6175 – 9 – 1625	543	Ox4	41:68:27	wg4
Camp081	H	2017	6175 – 9 – 1625	543	Ox4	41:68:27	wg4
Camp084	MA	2017	6175 – 9 – 1625	543	Ox4	41:68:27	wg4
Camp080	H	2017	6175 – 9 – 1625	543	Ox4	41:68:27	wg4
Camp085	H	2018	6175 – 9 – 1625	543	Ox4	41:68:27	wg4
Camp086	V	2018	6175 – 9 – 1625	543	Ox4	41:68:27	wg4
Camp087	H	2018	6175 – 9 – 1625	543	Ox4	41:68:27	wg4
Camp089	H	2018	6175 – 9 – 1625	543	Ox4	41:68:27	wg4
Camp088	H	2018	6175 – 9 – 1625	543	Ox7	2724:68:27	wg14

In the column Isolation Source, H refers to clinical samples, MA to mammals (cattle and sheep), V to poultry and SW to surface waters.

ST, Sequence Type; CT, Complex Type; MLST, Multi Locus Sequence Typing; cg, core genome; wg, whole genome.

### Concordance Between the Typing Methods

This analysis was performed on the 93 strains selected in the panel (Methods 3.2 and **Supplementary Data S2**). The cgMLST INNUENDO, the cgMLST Oxford, and the wgMLST INNUENDO had an AWC of 1.000 to the cgMLST SeqSphere+ schema, meaning that all strains clustering together using one of these three typing schemes are also classified together with the cgMLST SeqSphere+. The cgMLST Oxford had an AWC of 0.948 with the cgMLST INNUENDO and, conversely, the cgMLST INNUENDO had an AWC of 0.956 with cgMLST Oxford; 95% of the strains are clustered similarly using either cgMLSTs Oxford and INNUENDO. The majority (93.7% and 94.5%) of the strains that clustered with the cgMLST SeqSphere+ schema were also grouped by the cgMLST INNUENDO and the cgMLST Oxford, respectively. The wgMLST INNUENDO bundled 94.0% of the strains in a similar manner as the cgMLST INNUENDO and 99.8% as the cgMLST Oxford (**Table 7**).

**Table 7:** Adjusted Wallace coefficients values (CI 95%) for typing schemes comparison.

	Extended MLST	cgMLST SeqSphere+	cgMLST INNUENDO	cgMLST Oxford	wgMLST INNUENDO
Extended MLST		1.000 (1.000–1.000)	0.931 (0.832–1.000)	0.935 (0.876–0.994)	0.757 (0.647–0.867)
cgMLST SeqSphere+	0.795 (0.637–0.953)		0.937 (0.843–1.000)	0.945 (0.895–0.994)	0.728 (0.596–0.859)
cgMLST INNUENDO	0.790 (0.630–0.951)	1.000 (1.000–1.000)		0.956 (0.909–1.000)	0.729 (0.597–0.862)
cgMLST Oxford	0.787 (0.622–0.952)	1.000 (1.000–1.000)	0.948 (0.852–1.000)		0.769 (0.634–0.903)
wgMLST INNUENDO	0.829 (0.658–0.997)	1.000 (1.000–1.000)	0.940 (0.828–1.000)	0.998 (0.996–1.000)	

## Discussion

From our long-term surveillance of campylobacteriosis at national scale, our data suggested the presence of recurring genotypes defined by an extended MLST method indexing 9-loci over a 13-year period. This study investigated the relationship of a collection of isolates classified in four commonly identified lineages in Luxembourg at genome level. The aim was to assess the potential occurrence of stable genomes through the concordance of different WGS-based typing schemes exploring and comparing isolates at the core genome level (cgMLSTs from SeqSphere+, Oxford, and INNUENDO) or at the pan genome scale (wgMLST INNUENDO).

Our findings suggested that the genetic population structure of *Campylobacter jejuni* is partly composed of clonal expansion of some genotypes that persist over a long period spanning up to 13 years. Contrary to the epidemic curve commonly detected in case of foodborne outbreaks, stable genetic lineages of this pathogen could emerge to observable frequency through an endemic pattern, causing human infections on a regular basis throughout the country. In order to delineate these lineages with more confidence, efforts were focused on (i) comparing data with a robust design, and (ii) defining cut-offs values aligned with previously published data from genetic variability of the species as well as pre-established threshold for the different typing schemes tested.

Our first concern was to avoid biases generated due to low quality in the raw data and/or assemblies by establishing defined criteria before applying the gene-by-gene approach (Clark et al., 2016; Cody et al., 2017; Llarena et al., 2018; Besser et al., 2019). Quality filtering is a key prerequisite for faithful comparison of genomic data and applied criteria should be clearly stated in all WGS related reports. In their studies, Cody et al. (2013) and Kovanen et al. (2014) implemented a quality threshold in filtering the length of the reads with fixed criteria before the assembly. In 2017, Cody et al. applied a maximum of 150 contigs covering at least 95% of cgMLST targets (Cody et al., 2017), whereas the INNUENDO pipeline included a QC step requiring an assembled depth of coverage of 30x associated with at least 98% of scheme targets found in the cgMLST analyses (Llarena et al., 2018). The Draft Standard of International Standardization Organization (ISO/DIS 23418) suggests a depth of coverage of at least 20x for Illumina short-read raw data and 95% of the read lengths should be over 120 bp (International Organization for Standardization) depending on the application. In our analyses, we implemented stringent criteria for quality filtering to ensure robustness and minimizing potential biases related to missing targets generated by poor quality sequencing data.

While a core genome of a bacterial species is expected to consist of a conserved panel of functional genes (also properly called housekeeping genes), mostly present in the genomes of interest and essential to the microorganism, the cgMLST of the three tested methods included a different number of loci. This discrepancy resulted from a more or less stringent definition of the core genome applied to a panel of reference genome varying in size and quality. It is also noteworthy that the cgMLST INNUENDO schema was specifically determined from the *C. jejuni* species while the two others have included some *C. coli* genomes to create their schemes. In summary, SeqSphere+ and INNUENDO selected targets present in at least 90% of the complete genomes (N = 12) or in 99.9% of draft genomes (N = 6,526), respectively (Llarena et al., 2018). The cgMLST Oxford was built from loci occurring in 95% of the *Campylobacter* sp. reference panel (N = 2,472) to take into consideration variation in sequence quality and applied algorithms (Cody et al., 2017). They proposed a more relaxed core genome definition as some isolates may contain mutations, leading to the reduction of the core genome size as more isolates are selected, and that analyses conducted on incomplete draft genomes might constitute a source of missing data (Cody et al., 2017). The finalized cgMLST Oxford scheme represents thus 82% of the reference genome NCTC11168, which places this typing scheme as an intermediate between a core genome and a whole genome MLST scheme with a total of 1,343 loci vs. 637/678 for the two others. A sample-set independent approach was recently proposed to select a conserved-sequence genome as a novel core genome methodology to address this issue (Van Aggelen et al., 2019).

Further, the locus definition is different in the various schemes as well as allele calling algorithms. In the so-called gene-by-gene approach, a locus does not necessarily correspond to the complete coding sequence of a gene but can constitute a specific region. Thus, each schema includes target sequences of varying length ranging from 100 bp to several kb. Surprisingly, the sizes distributions of the targets in the three cgMLSTs tested are very similar with approximately: 21% below 500 bp, 40% ranging from 500 to 1,000 bp, 26% ranging between 1,000 and 1,500 bp, and 13% above 1,500 bp. Except for the SeqSphere+ commercial platform, the design of allele-calling pipelines from the two others WGS-based schemes were published (Jolley et al., 2018; Silva et al., 2018). Both define alleles from sequence assemblies but perform a search by using nucleotide or translated sequences with BLASTN or BLASTP queries and by using “exemplar alleles” as reference or all alleles already recorded in the database. The procedure differs mainly when new sequences display no exact match with known alleles. However, both pipelines validate the nucleotide sequence after translation of DNA codons and include a threshold in percentage sequence identity and length.

A predefined allele distance threshold allows assignment of an unique identifier to genomes displaying a high level of similarities in their cg/wgMLST profiles. The cut-off distance value for distinguishing clusters is expressed as a number of ADs and is species or even lineage-specific. To calibrate this value, a test population commonly includes clonal outbreak strains as well as non-epidemiologically linked outgroups. Therefore, the established thresholds are based on strains collected over a relatively short period, and may thus not be appropriate for long-term surveillance. Genomic variations linked to insufficient sequencing quality and microevolutions generated during the gut passage are taken into account for classifying strains (Cody et al., 2013; Revez et al., 2013; Thomas et al., 2014; Barker et al., 2020). For instance, Cody et al. (2013) observed between 3 to 14 loci differences (of 1,643 loci in total) in *Campylobacter* sp., during human gut passage, mainly restricted to insertions and deletions in homopolymeric tracts in contingency loci regulating phase variations of surface structures (Jerome et al., 2011; Barker et al., 2020). To classify related-genomes, Cody et al. (2013) tested two methods: a hierarchical approach based on an increasing number of loci in order to detect closely related isolates and a pairwise comparison based on 1,026 loci shared by the 379 C*. jejuni* genomes analyzed. Their results lead to the conclusion that the hierarchical approach is better suited to examine isolates epidemiologically related, while pairwise comparisons are preferable for the identification of outbreaks without initial suspicion (Cody et al., 2013). We assessed genomic clusters in our WGS data with goeBURST and we found that defined low cut-off values ranging from 6 to 11 AD and from 5 to 9 AD were appropriated to classify profiles generated with cgMLST Oxford and wgMLST INNUENDO schemes, respectively. By utilizing our newly established thresholds, the classification was consistent with the ones created by cgMLST methods that use a predefined threshold like SeqSphere+ (AD = 13 of 637 targets) and cgMLST INNUENDO (AD = 4 of 678 targets).

Overall, a high concordance in clustering strains was observed between the three cgMLST typing schemes, although congruence is higher between the cgMLSTs Oxford and INNUENDO schemes (predictive of each other in 95% of the cases) compared to the SeqSphere+ scheme. This was not expected, at first glance, as cgMLST schemes from SeqSphere+ and INNUENDO have a close number of targets (637 vs. 678 targets, respectively) and share 68% of loci. The concordance between the cgMLST schemes Oxford and INNUENDO, both defined from a large collection of strains, suggests a more representative and stably defined core genome. It is noteworthy that in this study, the added value of the number of loci in the cgMLST Oxford cannot be truly attributed on its discriminative power as the datasets contain several clonal population. A largest test population, reflecting the genetic diversity within the *C. jejuni* species, would have been more appropriate for evaluating the resolution of the different typing schemes. As expected, cgMLST profiles could not be mapped with confidence to the wgMLST INNUENDO profiles including a significant larger number of targets. Differences in the accessory genome composition or in the allelic variations could explain these discrepancies. As all the lineages selected for this study originated from various hosts, it could be interesting to further investigate on a possible link between accessory genomes and niche adaption (Woodcock et al., 2017).

The clonality signal appearing through the concordance of the different typing schemes in classifying strains supports the idea of stability of these clones over time and sources. Two independent studies introduced the concept of monomorphic genotypes for *C. jejuni* within the generalist lineages Clonal Complex (CC) ST-21 (Wu et al., 2016) and ST-45 (Llarena et al., 2016). The first study investigated the genetic basis responsible for the hyper virulence of a known clone named “sheep abortion” (clone SA, ST-8), causing foodborne illnesses in human and ruminant abortion (Wu et al., 2016). The second study explored the population structure of the generalist ST-45-CC, overrepresented in human cases in Finland (Llarena et al., 2016). Considering another field, clonal expansion linked to the acquisition of antibiotic resistance has also already been highlighted in *Campylobacter* (Wimalarathna et al., 2013). Observing stable genotypes in *Campylobacter jejuni* over time are in accordance with these results, hypothesizing that predominant clonal evolution is a major adaptive evolutionary strategy in microbial pathogens (Tibayrenc and Ayala, 2017).

In our study, the best example for stable genome over time is lineage A (ST19-*gyrA*8-*porA*7) as its recurrence occurs over more than a decade, although at a low level, representing an average of 13.4% of human cases per year (data not shown). Thirty-two strains of 34 from diverse sources (human, cattle and sheep, poultry, and environmental samples) were gathered in the same genetic profile at the whole genome level. This result reflects that this lineage is likely derived from one common ancestor, which thereafter disseminated broadly to a variety of mammals and birds, clearly demonstrating an ability to disperse in the environment and adapt to different ecological niches. Thus, the question of the environmental transmission routes arises, particularly concerning animal reservoirs such as poultry and ruminants that could contribute to water contamination (Mughini-Gras et al., 2016). Persistent strains have already been identified, mainly in poultry farms and in milk, and it would be interesting to link lineage A with other contamination sources such as insects, rodents, drinking water, or the surrounding environment (Kudirkienė et al., 2010; Perez-Boto et al., 2012; Rauber-Würfel et al., 2019; Jaakkonen et al., 2020).

The lineage B (ST2254-*gyrA*9-*porA*1) arose unexpectedly from our national surveillance with an epidemic curve between March and April 2014 (>70 campylobacteriosis cases). Interestingly, after this episode, clinical isolates of this lineage were still collected but at a much lower frequency during the following four years. To put things into context, this particular ST was singular in 2014 and by querying the pubmlst.org database (Jolley et al., 2018); only a dozen strains had been recorded at that time including two from poultry origin. Interestingly, the same “clone” was finally isolated in the framework of the official controls conducted by the state veterinary laboratory in Luxembourg and supported chicken as a possible source of this outbreak. In molecular epidemiology, the expression “clone” generally refers to a set of independently isolated microbial organisms that have similar genotypic traits as a results of a shared common ancestor (Van Belkum et al., 2007). The analysis using different typing schemes gathered 80% of the tested strains from lineage B in the same CT, whereas only 50% of isolates from lineage C formed a cluster. These data support the occurrence of the most large-scale outbreak caused by *C. jejuni* ever identified in Luxembourg and linked to chicken imported from neighboring countries, as the local production is negligible. Two years after the epidemic episode, this clone was isolated from a bovine source for the first time, while the remainder of lineage B was mainly isolated from poultry. The extent of ecological niches suggests that strains from lineage B were able to cross ecological barriers and disseminate in the environment with a generalist profile (Sheppard et al., 2014).

Lineage C (ST464-*gyrA*8-*porA*1678) displayed two micro-epidemic peaks: one in March 2016 and a second in January and February 2017. Since then, its incidence has been low with less than 10 human cases per year since March 2017 and we observed a first sample of bovine source isolated in August 2017. An average of two human cases per month from December 2016 to May 2018 indicates the profile of an emerging clone tending to have an endemic profile. Notably, this lineage displays the *gyrA* allele 8, one of the nucleotide allele in *C. jejuni* containing the C257T mutation (i.e., the peptide shift Thr86Ile) which confers quinolone resistance (Ragimbeau et al., 2014). Indeed, dispersion of antimicrobial resistant lineages due to positive selection was previously described for bacterial pathogens, such as uropathogenic *Escherichia coli* (ST 131 for example) (Totsika et al., 2011; Yamaji et al., 2018) and *C. jejuni* (ST 464 for instance) (Cha et al., 2016). For lineage D (ST6175-*gyrA*9-*porA*1625), the first isolate was identified in 2012 from a human infection, then in 2014 and at the beginning of 2016. A link with poultry source was observed.

Whatever the typing scheme used, clear signals appeared in our molecular surveillance for identifying an outbreak (lineage B in 2014) or the phenomenon of recurrent clones, which cause of more than 50% of human infections in Luxembourg. This study provides new insights for the genomic surveillance of *Campylobacter* infections. Through the exploration of the large collection of data that we have initiated 15 years ago, we seek to demonstrate the strong interest in monitoring genotypes causing gastroenteritis in the sense that campylobacteriosis is not only of sporadic nature. A recent study based on collected WGS data in Denmark also supported these findings (Joensen et al., 2018).

Molecular surveillance of foodborne pathogens is currently implemented for *Salmonella* (Dangel et al., 2019), *Listeria* (Van Walle et al., 2018), and VTEC (Joensen et al., 2014) at the EU level (ECDC, 2019) and in the USA (Ribot et al., 2019). For *C. jejuni*, such monitoring in routine is hindered by the absence of a validated scheme at international level and the lack of evidence for the spread possibility of cross-border genotypes. The presence of recurring genotypes highlights the possible long-term existing of stable clones representing a risk factor of geographic spread that needs to be investigated further. Like for the acquisition of antibiotic resistance, persistent strains may have acquired specific phenotypic traits to adapt to other hosts or disperse in the environment. Habituation to ambient air (Rodrigues et al., 2015; Rodrigues et al., 2016), adhesion to inert surface (Sulaeman et al., 2010; Oh et al., 2016) and biofilm formation (Reuter et al., 2010; Turonova et al., 2015) could contribute to the survival strategies of *C. jejuni* in the environment. In the future, studying the phenotypic traits of recurrent clones and their relationship to spatiotemporal persistence would broaden our understanding on *Campylobacter* adaptation and its transmission to humans.

## Data Availability Statement

Sequenced raw reads have been uploaded to ENA and are available under the accession project number PRJEB40465.

## Author Contributions

CR conceived, designed, and overseen the study. OT contributed to data analyses, data organization, and the conception of the paper. MN and CR were involved in data acquisition. A-KL performed all INNUENDO related analyses. MH conducted the targets comparison analysis. CP and SL provided environmental and veterinary samples, respectively. JM advised on bioinformatics analysis and revised the manuscript. MN wrote the initial draft of the manuscript. All co-authors critically revised the manuscript. All authors contributed to the article and approved the submitted version.

## Funding

This work is a part of the CampylOmic project funded by the National Research Fund of Luxembourg (C17/BM/11684203). MN’s thesis belongs also to the project RFI Food for Tomorrow of Région Pays de la Loire in France (RFI N°00002087).

## Conflict of Interest

The authors declare that the research was conducted in the absence of any commercial or financial relationships that could be construed as a potential conflict of interest.
