# Supplementary material for: Investigating Major Recurring Campylobacter jejuni Lineages in Luxembourg Using Four Core or Whole Genome Sequencing Typing Schemes
Source: Front Cell Infect Microbiol. 2021 Jan 8;10:608020. doi: 10.3389/fcimb.2020.608020 (PMC7819963; doi:10.3389/fcimb.2020.608020)
Supplement: Supplementary file 1 [file Table_1.docx]

**cgMLST target definition.** To determine the core genome MLST (cgMLST) gene set, we performed a genome-wide gene-by-gene comparison using the cgMLST Target Definer function version 1.0 of the SeqSphere+ software (Ridom GmbH, Münster, Germany) with the same default parameters as described previously except that a sequence identity of ≥80% was applied here (1). The genome of the *C. jejuni* strain NCTC 11168/ATCC 700819 (NC_002163.1, 22-JUL-2013) served as seed genome. Twelve other finished RefSeq *Campylobacter* genomes (ten *C. jejuni* and two *C. coli*; NC_003912.7, NC_008787.1, NC_009707.1, NC_009839.1, NC_014802.1, NC_017279.1, NC_017280.1, NC_017281.1, NC_018521.1, NC_018709.2, and NC_022132.1, NC_022347.1) covering the species variability as defined by MLST functioned as penetration query genomes. From the resulting core genome targets 44 genes were manually moved to the accessory genome targets as they could not be detected in more than 90% of the 49 *C. coli* genomes described by Cody *et al.* (2). Thus, the final ‘hard defined’ cgMLST scheme consisted of 637 genes. In addition, are 958 accessory genome MLST targets part of the scheme. The scheme was evaluated with 34 Illumina sequenced genomes from 3 focal outbreaks and sporadic case isolates (3) and repeat sequencing genomic data from same DNA and within-patient genomic data from isolates with the same ST (2). Using those results a complex type (CT) threshold of thirteen was defined to give guidance for delineation of possibly related from not-related genomes (1). The detailed description of the genomes and parameters used for scheme definition as well as all core and accessory genome genes including all allele variants can be found at www.cgMLST.org.

1. Ruppitsch W, Pietzka A, Prior K, Bletz S, Fernandez HL, Allerberger F, Harmsen D, Mellmann A. Defining and evaluating a core genome multilocus sequence typing scheme for whole-genome sequence-based typing of *Listeria monocytogenes*. J Clin Microbiol. 2015 Sep;53(9):2869-76. PubMed PMID: 26135865

2. Cody AJ, McCarthy ND, Jansen van Rensburg M, Isinkaye T, Bentley SD, Parkhill J, Dingle KE, Bowler IC, Jolley KA, Maiden MC. Real-time genomic epidemiological evaluation of human *Campylobacter* isolates by use of whole-genome multilocus sequence typing. J Clin Microbiol. 2013 Aug;51(8):2526-34. doi: 10.1128/JCM.00066-13. PubMed PMID: 23698529

3. Mellmann A, Mosters J, Bartelt E, Roggentin P, Ammon A, Friedrich AW, Karch H, Harmsen D. Sequence-based typing of flaB is a more stable screening tool than typing of flaA for monitoring of *Campylobacter* populations. J Clin Microbiol. 2004 Oct;42(10):4840-2. PubMed PMID: 15472357
